# Predictive Factors of Persistent Growth Hormone Deficiency and Impact on Final Height

**DOI:** 10.3390/children12030324

**Published:** 2025-03-03

**Authors:** Flavia Urbano, Mariangela Chiarito, Luigi Antonio Moscogiuri, Crescenza Lattanzio, Rossella Vitale, Orazio Valerio Giannico, Gabriele Annesi, Clara Zecchino, Maria Felicia Faienza

**Affiliations:** 1 Pediatric Hospital “Giovanni XXIII”, 70126 Bari, Italy; flavia.urbano@policlinico.ba.it (F.U.); mariangela.chiarito@policlinico.ba.it (M.C.); luigi.moscogiuri@studenti.uniba.it (L.A.M.); c.lattanzio8@studenti.uniba.it (C.L.); r.vitale7@studenti.uniba.it (R.V.); g.annesi1@studenti.uniba.it (G.A.); clara.zecchino@policlinico.ba.it (C.Z.); 2 Unit of Statistics and Epidemiology, Local Health Authority of Taranto, 74121 Taranto, Italy; 3 Pediatric Unit, Department of Precision and Regenerative Medicine and Ionian Area, University of Bari “Aldo Moro”, 70124 Bari, Italy

**Keywords:** predictive factors, persistent GHD, final height, transition phase, retesting

## Abstract

Background/Objectives: Recombinant growth hormone (rhGH) treatment plays an important role in the transition phase in those subjects diagnosed as having persistent growth hormone deficiency (GHD). We aimed to identify the main predictors of persistent GHD in a large cohort of subjects with childhood-onset GHD who underwent retesting and their correlation with height gain and mid-parental height (MPH). Methods: Anthropometric data, such as growth rate; bone age (BA); IGF-1 SDS at the start, at 1 year, and at the end of rhGH therapy; GH peak at diagnosis and at retesting; brain Magnetic Resonance Imaging (MRI) at diagnosis; and height gain upon reaching final height (FH) and compared to MPH, were obtained from medical records of GHD patients. Results: Persistent GHD was detected in 37 out of 91 (40.7%) GHD subjects. In univariate analysis, persistent GHD was associated with growth rate at 1 year (*p* = 0.0117) and with the first test GH peak (*p* = 0.0290). In the regression analysis, persistent GHD was positively associated with growth rate at 1 year (*p* = 0.0294) and negatively with female gender (*p* = 0.0424). Height gain was positively associated with growth rate (*p* = 0.0010) and with age at onset (*p* = 0.0021), while an inverse association with BA at baseline (*p* = 0.0002) and IGF-1 SDS (*p* = 0.0321) was found. Conclusions: Our study confirmed that the most important predictor of persistent GHD is the growth rate in the first year of therapy. Furthermore, growth rate in the first year, female gender, and lower BA at diagnosis are predictors of rhGH efficacy both in terms of height gain and target height achievement.

## 1. Introduction

The role of recombinant growth hormone (rhGH) treatment in children diagnosed with growth hormone deficiency (GHD) is well established [1,2,3]. The efficacy of rhGH replacement therapy is evaluated on the increase in the linear growth rate, and on the achievement of an adult height in line with the genetic potential [4].

Indications for rhGH therapy also include some categories of patients without GHD, such as small-for-gestational-age (SGA) children without catch-up growth, Noonan syndrome [5,6], Turner syndrome [7], Prader–Willi syndrome, SHOX haploinsufficiency, and chronic renal failure. Furthermore, the growth hormone (GH) also plays an important role in the transition phase, in particular in reaching peak bone mass and influencing body composition, metabolic profile, maturation, and gonadal function [8]. The transition phase begins with the achievement of the final height (FH), defined by a growth rate <1.5–2 cm/year or a bone age (BA) of 14–15 years in females and 16–17 years in males (Tanner stage 5), and it ends when the peak bone mass is reached, which occurs at an average age of 23.1 years in males and 19.9 years in females [8,9]. Retesting of all subjects who have undergone rhGH replacement therapy is recommended in order to distinguish between transient and persistent GHD. Retesting should be performed at least one month after rhGH discontinuation to properly determine GH physiological secretion [10].

The diagnosis of GHD both in childhood and in the transition phase in Italy is regulated by specific clinical, biochemical, and neuroradiological criteria codified by the Italian Medicines Agency (AIFA) Note 39 [11]. There are several GH stimulation tests whose diagnostic accuracy depends on the cut-off values used [11,12]. The gold standard recommended for both the diagnosis and retesting of GHD is the insulin tolerance test (ITT), whose cut-off GH values are ≤8 μg/L and ≤6 μg/L, respectively [13]. In case of contraindications to the execution of the ITT, as in patients with a history of seizures, epilepsy, cardiovascular or cerebrovascular diseases, arginine, clonidine, or glucagon, stimulation tests can be used for the diagnosis of GHD (cut-off value ≤ 8 μg/L), while the combined test with the GH-releasing hormone plus arginine (GHRH + A) can be used for retesting GH secretion [14]. Different GH cut-off values have been proposed for the combined GHRH + A stimulation test. However, the cut-off GH peak commonly considered diagnostic is ≤19 μg/L, although it has a variable diagnostic accuracy depending on the underlying pathological condition (congenital GHD, isolated GHD, multiple adeno-hypophyseal deficits, cancer survivors) or weight [15,16]. The combined test is not recommended in idiopathic GHD or in subjects undergoing cranial irradiation for acute lymphoblastic leukemia during childhood due to the risk of false positives and in congenital GHD with hypothalamic-pituitary abnormalities due to the risk of false negatives [12,15,16].

Recently, a GH peak to a glucagon stimulation test (GST) < 5.8 μg/L has been demonstrated to represent an accurate diagnostic cut-off for young adults with childhood-onset GHD and a high probability of permanent GHD [17].

The importance of restarting rhGH replacement therapy in cases with persistent GHD is well recognized, and the main predictor of persistent GHD seems to be the lower GH peak at diagnosis [10,18]. However, other predictors of persistent GHD have not yet been clearly identified.

Starting from this consideration, the primary aim of this study was to define the main predictive factors of persistent GHD in a large cohort of patients diagnosed with idiopathic and isolated GHD in childhood. The secondary aim was to evaluate the association between the persistence of GHD and the response to rhGH therapy in terms of height gain upon reaching FH and compared to MPH.

## 2. Materials and Methods

In this retrospective study, we included data obtained from medical records of subjects diagnosed as affected by idiopathic and isolated GHD in childhood who underwent retesting in the period from 2012 to 2024. Height measurements, expressed as height standard deviation scores (HSDS), were evaluated at the beginning of treatment, at 6 months, at 12 months, and at the end of rhGH therapy and compared with the standard growth charts for the Italian population [19]. The BA for each key time point and pubertal stages at the start assessed with the Tanner method [20] were also evaluated.

Growth velocity (GV) (cm/year, SDS) was obtained at every time point as the ratio between the difference in height and the difference in age with respect to the previous time point, and evaluated on Tanner charts [21].

FH was defined as the height measured when the GV was below 1 cm/year and when epiphyseal closure occurred on the wrist radiograph. Target height (TH) was calculated as MPH plus 6.5 cm for males and minus 6.5 cm for females.

For each patient, the following data were also collected: rhGH dose at the start of treatment, at 6 months, at 12 months, and at the end of therapy; MPH; height gain upon reaching final height (ΔFH-H at baseline) and compared to MPH (ΔFH-MPH); mean duration of rhGH therapy; type of stimulation test at diagnosis and at retesting; GH peak at diagnosis and at retesting; IGF-1 SDS at the beginning of treatment, at 6 months, at 12 months, and at the end therapy; and brain Magnetic Resonance Imaging (MRI) at diagnosis.

This study was approved by the Independent Ethics Committee of Policlinico of Bari, Azienda Ospedaliero-Universitaria Consorziale Policlinico, Bari, Italy. Informed consent has been waived because this study is a retrospective study.

### Statistical Analysis

Statistical analysis was performed using R version 4.2.3. Categorical variables were reported as absolute and relative frequencies and compared through the Fisher test. Normality was assessed with the Shapiro–Wilk test. Continuous variables were reported as median and IQR and compared through the Wilcoxon rank sum. Multivariable binary logistic or linear regression models were fitted in order to assess the effect of different variables on the evaluated outcomes, namely persistent GH deficiency, Δ(FH-MPH) SDS, and Δ(FH-baseline Ht) SDS (Gain SDS). For each outcome, we reported two models. Model 1 was the full model with all evaluated predictors; model 2 was the reduced model, including only the variables that were significant in model 1. We always included in both models the following variables: gender, baseline age, and baseline bone age to adjust for possible confounding. The sample size was idoneous, relying on the one-in-ten rule. For each linear model, a global validation linear model assumption significance test was performed to verify the linearity assumption of the dependent variable and the normality and homoskedasticity assumptions of the residuals. Logistic models were evaluated through the Aikake information criterion. Confidence intervals were reported for all models.

## 3. Results

Data from 91 subjects affected by childhood-onset idiopathic and isolated GHD (56 males and 35 females) were collected. General characteristics of the enrolled patients are reported in Table 1.

At baseline, the median age was 11.83 years, the median BA was 9.25 years, and the median IGF-1 SDS was −1.58. The mean treatment duration was 4.1 years, with a median dose of 31 μg/Kg/day. After 1 year of rhGH treatment, the median BA was 11.0 years. The mean growth rate at 1 year was 8.85 cm/year.

At diagnosis, as the first test, we used, alternatively, ITT, arginine, clonidine, or glucagon. The second test was performed to confirm GH deficiency in all patients with a GH peak response below the cut-off value at the first test. The median GH peak was, respectively, 4.2 µg/L at the first test and 5.28 µg/L at the second test. Retesting was performed with GHRH + A or ITT at least one month after rhGH therapy discontinuation, and the median GH peak was 10.3 µg/L for the subgroup retested with ITT and 21.4 µg/L for the subgroup retested with GHRH + A.

Brain MRI showed abnormalities of the hypothalamic-pituitary region in 14 out of 91 patients (15.4%). Persistent GHD was detected in 37 out of 91 patients, with an overall prevalence of 40.7%. Of the 37 subjects with persistent GHD, 28 (75.7%) were males and 9 (24.3%) were females (*p* = 0.0286). Stratifying the cohort based on brain MRI, persistent GHD was found in 6 out of 14 (42.9%) patients with MRI alterations (adenohypophysis hypoplasia, neurohypophysis ectopy or agenesis, thinning or lack of pituitary stalk, partial empty sella), and in 31 out of 77 (40.3%) patients without MRI alterations. However, there was not a significant difference between the two subgroups (*p* > 0.999).

In univariate analysis, persistent GHD appeared to be associated with growth rate at 1 year (*p* = 0.0117) and with GH peak at the first test (*p* = 0.0290).

The results of multiple logistic regression models for persistent GHD are reported in Table 2.

In the full model, persistent GHD showed a positive association with growth rate in the first year of treatment (OR 1.29, 95% CI 1.05–1.61, *p* 0.0194). Results of the reduced model, obtained after excluding non-significant predictors, showed a still positive association with growth rate at 1-year (OR 1.25, 95% CI 1.03–1.56, *p* 0.0294) and a negative association with female gender (OR 0.36, 95% CI 0.13–0.94, *p* 0.0424).

The results of linear regression models for Δ(FH-MPH) SDS are reported in Table 3.

In the full model, Δ(FH-MPH) SDS showed a positive association with growth rate at 1 year (Beta 0.09, 95% CI 0.01–0.18, *p* 0.0353). In the reduced model, an almost significant, positive association was still found between Δ(FH-MPH) SDS and growth rate (Beta 0.08, 95% CI 0.00–0.17, *p* 0.0507), and a negative association was found between Δ(FH-MPH) SDS and female gender (Beta −0.48, 95% CI −0.88;−0.09, *p* 0.0177).

The results of linear regression models for height gain Δ(FH-Ht at baseline) SDS are reported in Table 4. Δ(FH-baseline Ht) is referred to as Gain SDS. In the full model, Gain SDS showed a positive association with age at baseline (Beta 0.19, 95% CI 0.06–0.32, *p* 0.0040) and 1-year growth rate (Beta 0.10, 95% CI 0.04–0.16, *p* 0.0020) and a negative association with bone age at baseline (Beta −0.24, 95% CI −0.38;−0.11, *p* 0.0004) and with IGF-1 SDS (Beta −0.31, 95% CI −0.59;−0.03, *p* 0.0321). In the reduced model, Gain SDS is positively associated with age at baseline (Beta 0.20, 95% CI 0.08–0.33, *p* 0.0021) and 1-year growth rate (Beta 0.10, 95% CI 0.04–0.16, *p* 0.0010) and negatively associated with bone age at baseline (Beta −0.25, 95% CI −0.38;−0.12, *p* 0.0002).

## 4. Discussion

The identification of predictive factors of persistent GHD is of great interest in the field of pediatric endocrinology. In our cohort, we evaluated the possible association between auxological, biochemical, and instrumental examinations and the persistence of GHD upon rhGH therapy discontinuation.

As a second endpoint, we also analyzed the presence of a possible association between persistent GHD and the response to rhGH therapy in terms of height gain upon reaching final height and compared to the average family height.

In our cohort, persistent GHD was detected with a prevalence of 40.7%, in agreement with other epidemiological studies [22,23].

In about 85% of patients, we did not find abnormalities of the hypothalamic-pituitary region without any difference between transient and persistent GHD. Conversely, according to the previous literature, a statistically significant association was found between persistent GHD and anatomical abnormalities of the hypothalamic-pituitary region, suggesting that patients having organic GHD are predisposed to permanent GHD [24,25]. The reason for this discordance could be attributable to the poor diagnostic reliability of the stimulus (GHRH + A) used for retesting in previous studies, as well as to the related cut-off values. Unlike in our study, ITT was the most used stimulation test. Currently, the gold standard for retesting is the ITT test, which has a greater diagnostic accuracy.

In our study cohort, a lower response to the first test was significantly associated with persistent GHD in the univariate analysis. This result was expected since the association between the lowest GH peak and the persistence of GHD was already known in the literature [18,25].

In addition, the growth rate after 1 year of therapy (cm/year) was significantly associated with persistent GHD in our study cohort (OR 1.29, 95% CI 1.05;1.61), and, therefore, it was a strong predictor of the persistence of GHD.

Moreover, a positive linear association between growth rate after 1 year of therapy and both height gain upon reaching final height (ΔFH-baseline Ht SDS) and compared to mid-parental height (ΔFH-MPH SDS) was found.

Therefore, the growth rate at 1 year correlates positively with the persistence of GHD, and it is also a predictive factor of a better response to therapy.

Applying the logistic regression model, the female sex presents an inverse correlation with the persistence of GHD. It is, therefore, reasonable to assume that the female sex represents a protective factor with regard to the persistence of GHD. Previous data in the literature did not demonstrate a significant gender difference between patients with persistent and transient GHD [24]. Moreover, applying the linear regression models, the female sex shows a negative association with ΔFH-MPH SDS. This evidence supports the hypothesis that the female sex is not only less predisposed to the persistence of GHD but also that it is closer to the target height, thus resulting in a predictive factor of a good response to therapy. In our study, we did not observe a statistically significant correlation between the variables age of onset or BA at baseline and persistence of GHD. This is the main limitation of the study and could be attributed to the poor reliability of the GHRH + A stimulation test used for retesting before the last update of AIFA Note 39. No correlation was even found in our study between IGF-1 and persistent GHD. This result was expected as, regardless of the presence or absence of GHD, IGF-1 levels are dependent on the nutritional status and can be influenced by different variables such as age, BMI, pubertal stage, physical fitness, glucocorticoid exposure, prolactin, and testosterone levels. For these reasons, IGF-1 is not a reliable biomarker of permanent GHD [26].

On the contrary, an inverse correlation between BA at baseline and height gain has been demonstrated. The inverse relationship between BA and lower height gain agrees with the scientific evidence accumulated over the years, which is also widespread in the clinical field, according to which a higher BA is predictive of a lower height prognosis. Interestingly, height gain is inversely associated with baseline IGF-1 SDS. This finding supports the crucial role of IGF-1 SDS as a prognostic factor for the child’s growth potential.

In conclusion, the most important predictor of persistent GHD is the growth rate in the first year of therapy; on the contrary, the female gender seems to be protective against GHD persistence. Furthermore, growth rate in the first year of therapy, female gender, and a lower BA at diagnosis are all predictors of the efficacy of rhGH therapy both in terms of height gain and target height achievement. Previous studies have already highlighted the importance of GH response during the first year of therapy as an indicator of the treatment efficacy as well as a predictor of the adult height outcome, allowing the early identification of non-responders to GH replacement therapy in whom treatment discontinuation might be considered [27,28].

Further studies are needed to establish clear diagnostic criteria for persistent GHD in order to identify candidates for continuous therapy during the transitional age.

## Figures and Tables

**Table 1:** General characteristics of the enrolled patients.

N = 91	Transient GHD	Persistent GHD	Wilcoxon Test	Total
	Median (IQR)	Median (IQR)	*p* Value	Median (IQR)
Age at baseline (y)	11.67 (10.50; 12.54)	12.50 (10.33; 13.92)	0.1503	11.83 (10.33; 13.21)
Bone age at baseline (years)	9.00 (7.50; 10.50)	10.25 (7.83; 11.00)	0.0813	9.25 (7.63; 10.75)
IGF-1 SDS baseline	−1.59 (−1.83; −1.37)	−1.59 (−1.71; −1.28)	0.3791	−1.58 (−1.82; −1.29)
Treatment duration (years)	4.17 (3.35; 5.88)	4.08 (3.02; 5.33)	0.5312	4.1 (3.17;5.75)
Treatment dose (µg/Kg/day)	31 (28; 32)	30 (28; 32)	0.8932	31 (28; 32)
Bone age at 1 year	11.00 (9.31; 11.81)	11.5 (9.75; 13.00)	0.1030	11.0 (9.5; 12.5)
Growth rate 1st year (cm/y)	7.9 (6.80; 9.23)	9.8 (7.70; 11.00)	0.0117	8.85 (7.20; 10.15)
1st test response (µg/L)	4.48 (2.45; 6.42)	3.50 (1.80; 4.20)	0.0290	4.2 (2.12; 5.77)
2nd test response (µg/L)	5.28 (3.73; 7.90)	5.28 (2.50; 7.07)	0.5047	5.28 (3.23; 7.75)
Retesting	3 (1)	3 (0)	0.3377	3 (0)
GH peak at retesting with ITT (µg/L)	12.8 (8.3; 18.7)	3.7 (1.1; 4.6)	0.0005	10.3 (5.8; 15.0)
GH peak at retesting with GHRH+A (µg/L)	40.3 (28.5; 52.4)	9.2 (6.4; 12.9)	<0.0001	21.4 (9.3; 41.0)

**Table 2:** Multiple binary logistic regression for persistent GHD.

Persistent GHD, Model 1	OR	95% CI	*p*-Value
Female sex	0.42	0.14; 1.14	0.0951
Age at onset	0.79	0.49; 1.23	0.3022
Baseline bone age	1.38	0.88; 2.24	0.1670
Baseline IGF-1 SDS	1.66	0.61; 4.61	0.3190
2nd test response	0.95	0.84; 107	0.3917
Treatment dose (µg/Kg/day)	1.00	0.90; 1.11	0.9756
Growth rate 1st year	1.29	1.05; 1.61	0.0194
Persistent GHD, Model 2			
Female sex	0.36	0.13; 0.94	0.0424
Age at onset	0.77	0.49; 1.19	0.2525
Baseline bone age	1.38	0.89; 2.21	0.1533
Growth rate 1st year	1.25	1.03; 1.56	0.0294

**Table 3:** Results of multiple linear regression for Δ (FH-MPH) SDS.

Δ(FH-MPH) SDS, Model 1	Beta	95% CI	*p*-Value
Female sex	−0.39	−0.83; 3.32	0.0723
Age at onset	−0.05	−0.24; 0.14	0.5852
Baseline bone age	−0.03	−0.22; 0.16	0.7721
Baseline IGF-1 SDS	0.13	−0.28; 0.55	0.5235
Response 2nd test	0.00	−0.05; 0.05	0.9909
Treatment dose (ug/Kg/day)	−0.01	−0.05; 0.03	0.6927
Growth rate 1st year	0.09	0.01; 0.18	0.0353
Δ(FH-MPH) SDS, Model 2	Beta	95% CI	*p* Value
Female sex	−0.48	−0.88; −0.09	0.0177
Age at onset	−0.05	−0.23; 0.14	0.6171
Bone age at baseline	−0.04	−0.23; 0.14	0.6588
Growth rate 1st year	0.08	−0.00; 0.17	0.0507

**Table 4:** Results of multiple linear regression for Gain SDS.

Gain SDS, Model 1	Beta	95% CI	*p*-Value
Female sex	−0.05	−0.25; 0.25	0.7345
Age at onset	0.19	0.06; 0.32	0.0040
Baseline bone age	−0.24	−0.38; −0.11	0.0004
Baseline IGF-1 SDS	−0.31	−0.59; −0.03	0.0321
Response 2 test	0.02	−0.01; 0.06	0.1841
Dose treatment (ug/Kg/day)	−0.00	−0.03; 0.03	0.8483
Retesting	0.04	−0.29; 0.37	0.8200
Growth rate 1st year	0.10	0.04; 0.16	0.0020
Gain SDS, Model 2	Beta	95% CI	*p* Value
Female sex	−0.03	−0.33; 0.26	0.7956
Age at onset	0.20	0.08; 0.33	0.0021
Baseline bone age	−0.25	−0.38; −0.12	0.0002
Baseline IGF-1 SDS	−0.25	−0.52; 0.02	0.0633
Growth rate 1st year	0.10	0.04; 0.16	0.0010

## Data Availability

The data presented in this study are available upon request from the corresponding author.
